# Plant immune receptors interact with hemibiotrophic pathogens to activate plant immunity

**DOI:** 10.3389/fmicb.2023.1252039

**Published:** 2023-10-09

**Authors:** Diao Zhou, Xingzhou Chen, Xinggang Chen, Yandong Xia, Junang Liu, Guoying Zhou

**Affiliations:** ^1^Key Laboratory of National Forestry and Grassland Administration on Control of Artificial Forest Diseases and Pests in South China, Central South University of Forestry and Technology, Changsha, China; ^2^Hunan Provincial Key Laboratory for Control of Forest Diseases and Pests, Central South University of Forestry and Technology, Changsha, China; ^3^Key Laboratory of Cultivation and Protection for Non-Wood Forest Trees, Ministry of Education, Central South University of Forestry and Technology, Changsha, China

**Keywords:** hemibiotrophic pathogen, cell surface pattern recognition receptor, intracellular immune receptor, effector, plant immunity

## Abstract

Phytopathogens pose a devastating threat to the productivity and yield of crops by causing destructive plant diseases in natural and agricultural environments. Hemibiotrophic pathogens have a variable-length biotrophic phase before turning to necrosis and are among the most invasive plant pathogens. Plant resistance to hemibiotrophic pathogens relies mainly on the activation of innate immune responses. These responses are typically initiated after the plant plasma membrane and various plant immune receptors detect immunogenic signals associated with pathogen infection. Hemibiotrophic pathogens evade pathogen-triggered immunity by masking themselves in an arms race while also enhancing or manipulating other receptors to promote virulence. However, our understanding of plant immune defenses against hemibiotrophic pathogens is highly limited due to the intricate infection mechanisms. In this review, we summarize the strategies that different hemibiotrophic pathogens interact with host immune receptors to activate plant immunity. We also discuss the significant role of the plasma membrane in plant immune responses, as well as the current obstacles and potential future research directions in this field. This will enable a more comprehensive understanding of the pathogenicity of hemibiotrophic pathogens and how distinct plant immune receptors oppose them, delivering valuable data for the prevention and management of plant diseases.

## 1. Introduction

Plant diseases have been a devastating threat throughout the history of agriculture. In addition to causing significant losses in global crop yields, plant diseases present major challenges to natural and agricultural systems. Phytopathogens cause devastating plant diseases by deploying infection strategies (Fisher et al., 2018). They can be classified into three main groups based on their infection strategies to extract plant nutrients: biotrophs, hemibiotrophs, and necrotrophs. Biotrophs extract nutrients from living cells and sustain host viability, whereas necrotrophs rapidly kill host cells to extract nutrients (Rajarammohan, 2021). Intermediate lifestyle hemibiotrophs begin in the biotrophic phase and subsequently transition to the necrotrophic phase (Damm et al., 2014). Hemibiotrophic pathogens (HPs) are prevalent and highly destructive phytopathogens that cause significant losses in crop quality and yield in key agricultural crops. The duration of each phase in HPs varies depending on factors such as the pathogen, host plant, temperature, secreted protein effectors, etc. (Qiu et al., 2022). For example, *Phytophthora infestans* has a shorter biotrophic phase than *Magnaporthe oryzae*, which has a shorter necrotrophic phase. Studies have also shown that hemibiotrophs utilize distinct effectors to adapt to various biotrophic/necrotic patterns. AVR3a stabilizes and targets the plant E3 ligase CMPG1 during the early stages of biotrophic infection by *P. infestans* to manipulate host immunity. AVR3a is subsequently downregulated, while the induction of other effectors INF1 and nep1-like proteins may facilitate the host transition to necrotrophic infection (Yaeno et al., 2011; Pirc et al., 2021). Although the term hemibiotroph was developed for pathogenic fungi, it is also sometimes used to describe the lifestyle of bacteria, oomycetes (Kraepiel and Barny, 2016; Panthapulakkal Narayanan et al., 2020). Different HPs differ in their pathogenic strategies, target hosts, etc. To induce disease in plants, HPs deploy various virulence factors to promote infection under defined environmental conditions.

**Figure 1:**
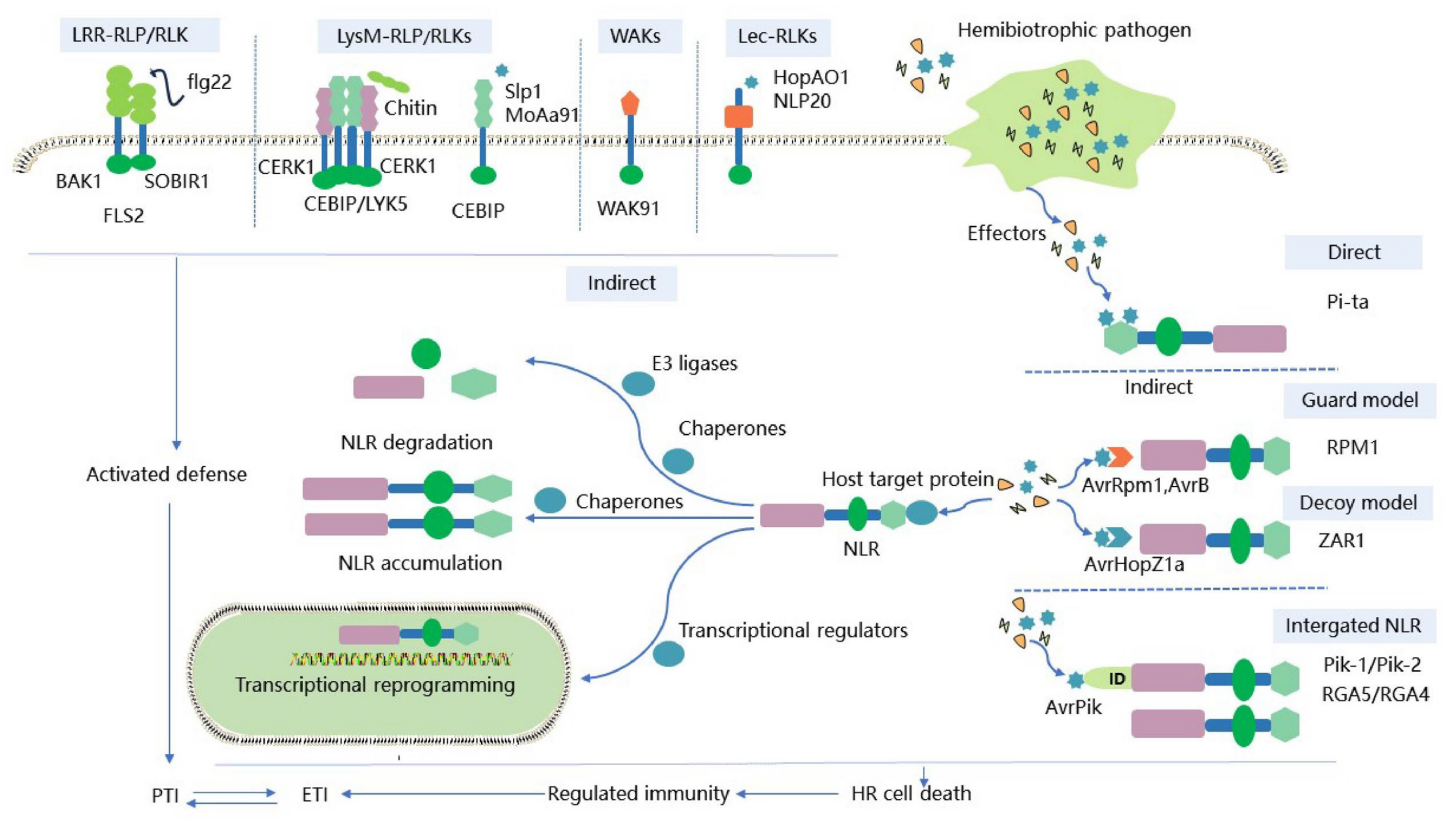
Schematic diagram of the two-layer immune system of plant immune receptors against HPs. Plant PRRs recognize PAMPs (e.g., bacterial flagellin , fungal chitin) or DAMPs (e.g., secretory minipeptides) to induce PTI. Adaptive HPs successfully translocate effectors into plant cells, thereby initiating a second round of plant immunity. Intracellular NLR immune receptors induce ETIs to trigger immune-related gene expression and local cell death through direct or indirect specific recognition of effector proteins. Activated PTIs enhance the defense response triggered by ETIs. While ETIs upregulate related genes that control signaling to PRRs, PTIs and ETIs together provide plants with strong immunity against HPs. ID, Integrated domain; PTI, PAMP-triggered immunity. ETI, Effector-triggered immunity; PRR, Pattern recognition receptor; HP, Hemibiotrophic pathogen; FLS2, Flagellin-sensitive 2; EFR, Elongation factor-Tu receptor; CERK1, Elicitor receptor kinase 1; CEBiP, Chitin elicitor binding protein; WAK, Wall-associated kinase; LRR, Leucine-rich repeat domain; LysM, Lysin motif domains; Lec, Lectin domain.

Plants have evolved a sophisticated surveillance system to protect themselves from HPs. It devotes resources and energy to growth and development without threat. However, when threatened by virulence factors secreted by phytopathogens, including toxins, phytohormones, and enzymes, plants rapidly regulate gene expression to protect the host from pathogens (Jones et al., 2016). The plant surveillance system mainly relies on two classes of immune receptors to detect pathogens: membrane-anchored pattern recognition receptors (PRRs) and intracellular nucleotide-binding and leucine-rich repeat receptors (NLRs; Dangl, 2013; Van de Weyer et al., 2019). In the classical zig-zag pattern, these two classes of receptor proteins correspond to the two layers of the plant immune system (Ngou et al., 2022). In the first layer of immune surveillance, PRRs recognize pathogen- or microbe-associated molecules (PAMPs or MAMPs, respectively) present in the extracellular space, which results in PAMP-triggered immunity (PTI) or MAMP-triggered immunity (MTI; Macho and Zipfel, 2014; Wang and Chai, 2020). However, many phytopathogens can manipulate host targets to inhibit PTI signaling and successfully deliver effectors to plant cells. In the second layer of host immune surveillance, intracellular NLRs activate effector-triggered immunity (ETI) by specifically recognizing intracellular pathogen effectors (Chen et al., 2012; Kourelis and van der Hoorn, 2018). The distinction between PTI and ETI is an excellent framework for explaining the plant immune system. ETIs enhance PTI-induced defense responses by altering the expression of key genes involved in PRR signaling elements transcription and translation. Conversely, PTI also enhances ETI-triggered defense responses. PTI and ETI work together to provide robust immunity to pathogens (Ngou et al., 2021; Yuan et al., 2021b). Over the past few decades, researchers have made significant progress in studying plant immune signaling controlled by PRRs and NLRs against necrotrophic and biotrophic pathogens. In this review, we summarize the strategies by which various HPs interact with host immune receptors to activate plant immunity (Figure 1). We focus on how various immune receptors perceive characteristic molecules from different HPs. We also discuss the commonalities and differences in the pathogenicity of different HPs. This may be crucial in explaining the potential threat of pathogens attack on the host for effective defense, it can also guide the improvement and breeding of genetically diseased crops.

## 2. Plasma membrane participates in plant immunity

The plasma membrane (PM) serves as the frontline of defense against pathogens in plants and is essential for pathogen detection, signal transduction, and cellular homeostasis maintenance. Several PRRs are present in plant PMs that detect PAMPs, DAMPs, or effector proteins that induces PTI response. In the zig-zag immune model, the PTI response prevents most HPs from invasion and reproduction. Adapted pathogens secrete large amounts of effectors to evade or inhibit PTI. Correspondingly, plants have also evolved intracellular NLR receptors that directly or indirectly recognize effectors and trigger a robust ETI response. This ultimately leads to localized plant cell death (Ngou et al., 2022). The co-resistance of PTI and ETI determines many plant defense responses to pathogen infection, such as protein phosphorylation, changes in ion flux, generation of reactive oxygen species (ROS), activation of mitogen-activated protein kinases (MAPK), and pathogenesis associated with cell wall strengthening (Tsuda and Katagiri, 2010). The activation of the MAPK signaling pathway by PRR at the PM results in the phosphorylation of target proteins in plant immunity. In *Arabidopsis*, the HP effector protein AvrRpt2 specifically inhibits the phosphorylation of MPK4 and MPK11 induced by the PM-localized receptor FLAGELLIN-SENSITIVE 2 (FLS2; Tsuda et al., 2013; Eschen-Lippold et al., 2016). It has also been shown that MPK3 and MPK6 can regulate malate metabolism to promote PM-mediated stomatal immunity during pathogen infection (Su et al., 2017).

Although HPs primarily activate the PM-anchored protein PRR, plants can also utilize lipids on the PM to sense HPs. These lipids trigger immune signals independent of PRR interactions. For example, the effector NLP of HPs is sensed by glycosphingolipids (GIPCs) on the PM, and it is speculated that a conformational changes in the GIPC-NLP complex induces pore formation in the PM and thus cell death (Mamode Cassim et al., 2018). In the basic defense process, recognition of PAMP or DAMP by the PRR of the PM induces cell wall modification that activates endocytosis of PRR and PAMP/DAMP, followed by degradation in the vacuole. This process initiates and amplifies immune signaling (Mbengue et al., 2016). For example, *Arabidopsis* RLCK BIK1, BSK1 interacts with FLS2 and is rapidly phosphorylated in an FLS2-dependent manner upon recognition of the bacterial flagellin peptide flg22. FLS2’s sustained anchoring to the PM is mediated *via* the BFA-sensitive endocytotic pathway under steady-state conditions (Beck et al., 2012). These findings contribute to a comprehensive understanding of the vital role of plant immunity in HP interference. Collectively, plant PM regulates immune responses by detecting HPs, activating signaling cascades, controlling the cellular entry and exit of molecules, and enabling PM endocytosis.

## 3. Plant surface immune receptors

Pattern recognition receptors (PRRs) play an important role in plant growth, development, reproduction, abiotic stress, and disease resistance, and many of them exhibit lineage-specific expansion to adapt to different pathogens within the innate immune system (Schellenberger et al., 2019; Ngou et al., 2022). Several plant PRRs have already been identified, such as FLS2, ELONGATION FACTOR-Tu RECEPTOR (EFR), ELICITOR RECEPTOR KINASE 1 (CERK1), and CHITIN ELICITOR BINDING PROTEIN (CEBiP; Tang et al., 2017). They recognize the bacterial flagellin epitope flg22, the EF-Tu epitope elf18, the plant elicitor polypeptide, and chitin released during pathogen infection, respectively (Chen et al., 2020). These PRRs form complexes with their corresponding ligands. These complexes activate downstream immune signals such as calcium influx, ROS production, MAPK signaling cascade, and defense responses (Couto and Zipfel, 2016; Zhou and Zhang, 2020). Based on the presence or absence of intracellular kinase domains, the PRR family is classified into receptor-like kinases (RLKs) and receptor-like proteins (RLPs), which act on the first layer of the plant immune system. RLKs consist of a variable N-terminal extracellular domain (ECD), a transmembrane region (TM), and a conserved cytoplasmic kinase domain (KD; Wang and Chai, 2020); In contrast, RLPs have only short domains and lack distinct kinase domains that require interaction with other kinase domain-containing proteins such as BAK1 and SOBIR1 to activate downstream signaling (Gust and Felix, 2014; Liebrand et al., 2014). RLKs and RLPs are classified into several subfamilies based on their ECDs, which include leucine-rich repeat (LRR) domains, lysin motif (LysM) domains, lectin (Lec) domains, and epidermal growth factor-like (EGF) repeat domains (Macho and Zipfel, 2014). Various subfamilies of PRRs exhibit commonalities and differences in detecting diverse HPs (Table 1; Figure 1).

**Table 1:** Overview of PRRs and the outcome of interactions with typical HPs to mediate plant immunity.

Plant receptor	Organism	Co-receptor	Pathogen	Effector	Outcome (Enhance or suppress the host defense response)
LRR					
FLS2	*Arabidopsis*	BAK1/BIK1	*P. syringae*	AvrPto	Suppress
FLS2	*Arabidopsis*	BAK1	*P. syringae*	HopB1	Suppress
FLS2	*Arabidopsis*	BAK1	*P. syringae*	HopQ1	Suppress
EFR	*Arabidopsis*	BAK1/SOBIR1	*P. syringae*	elf18	Enhance
RLP23	*Arabidopsis*	BAK1/SOBIR1	*P. syringae*	NLP20	Enhance
FLS2	*Arabidopsis*	BAK1	*P. syringae*	AvrPtoB	Suppress
**LysM**					
LYK5	*Arabidopsis*	CERK1	All	Chitin oligomers	Enhance
AtCERK1	*Arabidopsis*	LYK4/LYK5	All	Chitin oligomers	Enhance
CEBiP	rice	CERK1	All	Chitin oligomers	Enhance
CEBiP	rice	None	*M. oryzae*	Slp1	Suppress
CEBiP	rice	None	*M. oryzae*	MoAa91	Suppress
WAK					
OsWAK14/	Rice	None	*M. oryzae*	None	Enhance
OsWAK91	Rice	None	*M. oryzae*	None	Enhance
OsWAK92	Rice	None	*M. oryzae*	None	Enhance
OsWAK112d	Rice	None	*M. oryzae*	None	Suppress
OsWAK1	Rice	None	*M. oryzae*	None	Enhance
OsWAK25	Rice	None	*M. oryzae*	None	Enhance
AtWAKL10	Rice	None	*P. syringae*	None	Enhance
GmWAK1	Soybean	None	*P. infestans*	None	Enhance
SlWAK1	Tomato	None	*P. syringae*	None	Enhance
AtWAKL10	Tomato	None	*P. syringae*	None	Enhance
Lec					
FaMBL1	Strawberry	None	*C. fioriniae*	None	Enhance
OsLecRK	rice	None	*M. grisea*	None	Enhance
Pi-d2	rice	None	*M. grisea*	None	Enhance
LORE	*Arabidopsis*	None	*P. syringae*	HopAO1	Suppress
LecRK-IX.2	*Arabidopsis*	None	*P. syringae*	None	Enhance
LecRK-I.9	*Arabidopsis*	None	*P. syringae / P. infestans*	None	Enhance
SBP1/SBP2	*Arabidopsis*	BAK1/SOBIR2	*P. syringae*	NLP20	Suppress

^a^PRR, Pattern recognition receptor; HP, Hemibiotrophic pathogen; FLS2, Flagellin-sensitive 2, EFR, Elongation factor-Tu receptor; CERK1, Elicitor receptor kinase 1; CEBiP, Chitin elicitor binding protein; WAK, Wall-associated kinase; LRR, Leucine-rich repeat domain; LysM, Lysin motif domains; Lec, Lectin domain; *P. syringae*, *Pseudomonas syringae*; *M. oryzae*, *Magnaporthe oryzae*; *M. grisea*, *Magnaporthe grisea*; *C. fioriniae*, *Colletotrichum fioriniae*. ^b^None indicates that no data are available. ^C^This table is not a detailed list of all plant receptors for detecting hemibiotrophic pathogens (HPs).

### 3.1. LRR receptors sense HPs to confer plant immunity

Leucine-rich repeat-containing PRRs are the largest subfamily, including LRR-RLP and LRR-RLK family members. LRR-RLP/RLKs detect HPs by interacting with shorter ECD co-receptors of the same family to enhance immune signaling (Smakowska-Luzan et al., 2018). BRASSINOSTEROID INSENSITIVE 1 (BRI1)-ASSOCIATED RECEPTOR KINASE 1 (BAK1) is one of the most versatile co-receptors, also known as SOMATIC EMBRYOGENESIS RECEPTOR KINASES 3 (SERK3). It has only five LRRs in its ECD, which are centrally involved in various PTI signaling pathways (Wu et al., 2020). When hemibiotrophic bacteria interact with plants, a well-studied PRR is the LRR-RK FLS2 in most higher plants, which detects a 22-amino acid peptide derived from the N-terminus of bacterial flg22 (Lee et al., 2021). Recognition of flg22 by FLS2 and its co-receptor BAK1 is accompanied by rapid heterodimerization and phosphorylation, which activate plant immunity. To prevent the host immune responses, *P. syringae* secrete effectors to interfere with immune signals, such as AvrPto, AvrPtoB, HopB1, etc. AvrPto and AvrPtoB interact with FLS2 to prevent the formation of the FLS2-BAK1 complex and the phosphorylation of BIK1 (Gravino et al., 2017; Lei et al., 2020). HopB1 constitutively interacts with FLS2 prior to the activation of flg22. Upon activation, BAK1 is recruited to the FLS2-HopB1 complex. HopB1 cleaves BAK1 and its analogs via genetic transformation or bacterial delivery to inhibit FLS2 signaling and enhance pathogen virulence (Li et al., 2016; Wu et al., 2020). Like FLS2, another extensively studied LRR is the *Arabidopsis* EFR, which recognizes the N-terminal N-acetylated elongation factor Tu (EF-Tu) peptide of hemibiotrophic bacteria. Upon ligand binding, BAK1 is also recruited by EFR to participate in host immune signaling. Resistance to the hemibiotrophic bacteria *P. syringae* is activated by recognition of elf18C by EFR-*Cf*-9 in conjunction with SOBIR1 and BAK (Wu et al., 2019). In addition, rice LRR-RK XA21 can sense tyrosine sulfonate proteins derived from *Xanthomonas oryzae* to induce effective resistance to Xoo (Wei et al., 2016). These results suggest that under hemibiotrophic bacterial attack, plant LRR-RLKs participate in immune defense and self-development by phosphorylation upon binding to the corresponding co-receptors. Plant RLKs such as FLS2, EFR, and XA21 all belong to the LRR-XII subfamily, which suggests that RLKs of this subfamily may induce immunity by recognizing various protein ligands of hemibiotrophic bacteria. Similar to RLKs, BAK1 is also recruited to the two-component RLP or SOBIR1 complex by ligand recognition by RLP. BAK1 and SOBIR1 can transphosphorylate each other to activate immune signaling pathways. The *Arabidopsis* RLP23 forms a receptor complex by interacting with SOBIR1. Upon recognizing NLP20, it recruits the co-receptor BAK1 to the complex to enhance the LRR-mediated plant immune response to HPs (Albert et al., 2015; van der Burgh et al., 2019). Several effectors of hemibiotrophic bacteria, fungi, and oomycetes can prevent host LRR-RLP resistance to pathogens by inhibiting NLP-induced cell death, such as suppressor of necrosis 1 (SNE1) from *P. infestans* (Kelley et al., 2010), *Colletotrichum higginsianum* effector candidate (ChECs) from *C.higginsianum* (Kleemann et al., 2012), and MoNLP 1 (Chen et al., 2021), *M. oryzae* hypothetical effector gene 13 (MoHEG13; Mogga et al., 2016), and suppressors of plant cell death (SPDs) from *M. oryzae* (Sharpee et al., 2017). Regardless of whether hemibiotrophic bacteria, fungi, or oomycetes infect plants, although the immune response outcomes are different, FLS, EFR, and RLP23 all rely on kinase domain-containing proteins such as BAK1 or SOBIR1 receptor complexes for immune signaling. Different receptor complexes may elicit diverse immune responses by influencing intracellular structural domain phosphorylation and downstream immune signaling. Further studies of the phosphorylation properties of the different receptor complexes may reveal how these receptors are effective against localized HP infection.

### 3.2. LysM receptors sense HPs to confer plant immunity

During the game between HPs and plants, plant LysM ectodomains induce immune responses by recognizing N-acetylglucosamine molecules, such as fungal chitin oligomers and bacterial peptidoglycan (PGN; McCombe et al., 2022). The LysM family consists of LysM-RLK and LysM-RLP. LysM-RLKs have a LysM outer domain, a single channel transmembrane domain and a cytoplasmic kinase domain. LysM-RLP has only one outer domain connected to the outer membrane by GPI anchors (Buendia et al., 2018). CERK1 is a typical member of the LysM-RLK family consisting of three LysM structural domains. In rice and *Arabidopsis*, autophosphorylation of specific amino acids in CERK1 regulates chitin-induced immune signals secreted by HPs (Fliegmann et al., 2011; Suzuki et al., 2016; Yu et al., 2021). CERK1 not only recognizes hemibiotrophic fungal chitin, but is also a crucial co-receptor for bacterial PGN. CERK1, together with lysin motif receptor kinase 1 (LYM1) and LYM3, is required for the perception of bacterial PGN and for the development of basal resistance to *P. syringae*. Studies have also shown that deletion of CERK1 increases susceptibility to hemibiotrophic fungi and bacteria (Giovannoni et al., 2021). In *Arabidopsis*, LYK5 exhibits a greater affinity for chitin than CERK1. Once LYK5 externally detects chitin, CERK1 forms a dimer and transmits chitin signals to LYK5. Afterward, CERK1 phosphorylates LYK5 and itself in vesicles, which are then internalized (Erwig et al., 2017). LYK4 and AtCERK1 can form a complete complex with LYK5. LYK4 serves as a scaffolding protein or a co-receptor for LYK5, whereas AtCERK1 senses chitin and mediates homodimerization and phosphorylation, all of which promotes chitin triggering signaling (Wan et al., 2012; Cao et al., 2014; Xue et al., 2019). Unlike the chitin-sensing mechanism in *Arabidopsis*, the rice LysM-RLP CEBiP recognizes chitin and forms a dimer with OsCERK1 to activate plant disease resistance to HP and initiates downstream immune signaling pathways (Hayafune et al., 2014; Desaki et al., 2018; Hu et al., 2021). The results suggest that dimerization of the LysM receptor plays a vital role in ligand detecting, receptor activation, and immune signal transduction during HP infection. Aggregated PGN from hemibiotrophic bacteria is recognized as PAMP by the LysM receptor in *Arabidopsis* and activates immunity against hemibiotrophic bacteria in plants. Chitin is a major component of fungal cell walls. It plays a crucial molecular role in LysM-mediated host defense responses. Recognition and immune stimulation of chitin secreted by hemibiotrophic fungi in rice or *Arabidopsis* depend on the LysM-type PRRs OsCEBiP/OsCERK1, LYK4/LYK5, or AtCERK1, respectively. While plants use different sensing systems for bacterial-secreted PGN and fungal-secreted chitin, the chitin sensing system employed by fungi is structurally similar to the carbohydrate portion of bacterial-secreted PGN.

Plant LysM proteins recognize HPs to induce immune responses. HPs have evolved multiple mechanisms to evade plant immune recognition (Wang et al., 2022; Zhao L. et al., 2023). HPs secrete effectors with LysM domains that compete with high affinity for plant LysM receptors. Alternatively, HPs secrete effectors that modify plant LysM receptors to sequester, mask, alter, or prevent the host from degrading pathogen cell walls. These behaviors would regulate plant cytoplasmic signaling, suppress plant immunity, and regulate the transition of HPs from the biotrophic to the necrotrophic stage (Hu et al., 2021; Tian et al., 2022b). The secreted effector protein Slp1 of *M. oryzae* contains a LysM domain that accumulates at the early stage of infection between fungi and rice cells. This protein disrupts host chitin-triggered immunity by utilizing the LysM structural domain to competitively bind chitin oligosaccharides with CEBiP (Sanchez-Vallet et al., 2013). Similarly, *M. oryzae* depends not only on LysM Slp1 but also on MoAa91 of *M. oryzae*. This protein is vital for surface recognition and inhibition of chitin-induced plant immune responses. Further studies shown that MoAa91 competitively binds chitin to the rice immune receptor CEBiP to inhibit chitin-induced plant immune responses (Li et al., 2020). Many HPs-secreted LysM effectors remove chitin oligomers from the host infection site by intermolecular LysM dimerization, or prevent host recognition of chitin by the formation of polymeric precipitates, such as *C.higginsianum* ChElp1 and ChElp2 (Takahara et al., 2016; Sanchez-Vallet et al., 2020; Tian et al., 2022a). Intermolecular interactions such as homodimerization and phosphorylation demonstrate the significant implications of precisely management of host-pathogen processes, which are also essential for enhancing disease resistance in crops. However, it is unclear whether there are secreted proteins in HPs that more broadly regulate plant defense responses and cell death, potentially mediating the transition from biotrophy to necrotrophy.

### 3.3. Other PRR receptors sense HPs to confer plant immunity

Besides PRRs with LRR structures and LysM structures, there is increasing evidence that WAK receptors and lectin receptors play a significant role in plant-microbe interactions. A number of many immune-related WAKs have also been cloned recently. WAKs, a receptor-like kinase required for recognizing oligogalacturonides (OGs), often possess an extracellular EGF-like domain, and are found in both dicots and monocots (Stephens et al., 2022). They can identify pathogens with different lifestyles and regulate HP resistance by modifying host cell walls and regulating hormone fluctuations within host cells. WAKs play a crucial role in plant signaling pathways for immune and abiotic stress responses (Yue et al., 2022). OsWAKs in rice have been discovered to regulate the basic defense against *M. oryzae* either positively or negatively. Quantitative resistance has been positively affected by OsWAK14, OsWAK91, and OsWAK92, whereas resistance to rice blast has been negatively affected by OsWAK112d. OsWAK91 participates in intercellular signal transduction by generating ROS with possess antibacterial properties (Delteil et al., 2016). AtWAKL10 is thought to enhance plant resistance against *P. syringae*. Transgenic *Arabidopsis* lacking *WAK* exhibits increased susceptibility to *P. syringae* compared to the wild type (Bot et al., 2019). WAK receptors may regulate host immune resistance by modulating hormone fluctuations during HP infection of different host plants. Overexpression of *OsWAK1* and *OsWAK25* can enhance host resistance to *M. oryzae,* and salicylic acid (SA) treatment can up-regulate the expression of *OsWAK1* and *OsWAK25* genes (Li et al., 2009; Harkenrider et al., 2016). Recently, it was discovered that GmWAK1 relies on the SA pathway to alleviate oxidative stress-induced damage in soybeans resistant to *P. infestans* (Zhao M. et al., 2023). Immune-related WAK also prevents pathogen penetration during HP attack by altering cell wall composition to enhance cell wall strength. Upon infection of tomato by *P. syringae*, SlWAK1 strengthens the cell wall through callose deposition to restrict pathogen penetration and spread (Zhang et al., 2020). This ability of WAKs to regulate the cell wall indicates their potential role in plant growth, development, and response to abiotic stresses. OsWAK91/OsDees1 knockout rice has increased susceptibility to *M. oryzae* and inhibited growth (Delteil et al., 2016). In tomato, AtWAKL10 exhibits resistance to *P. syringae* while also upregulated when treated with the abiotic stress signaling factor S-nitroso-L-cysteine. Additionally, its knockout gene mutant displays increased tolerance to drought stress, but lower tolerance to salt stress (Bot et al., 2019). The WAK receptors not only enhance or inhibit host resistance when different HPs infect various hosts, but also affect plant growth and development. The mechanisms by which WAK receptors recognize or transduce pathogen signals and their impact on the transition of HPs from the biotrophic to necrotrophic stage remain unclear.

Lectin receptor-like kinases (LecRKs) are unique PRRs that specifically recognize carbohydrates such as mannose induced by elicitors or pathogens (De Coninck and Van Damme, 2022). LecRKs appear to constitute a vital recognition system on the surface of plant cells during plant-microbe interactions, and may play a vital role in plant immunity or stress responses (Wang and Gou, 2022). Furthermore, LecRKs are classified into L-type, C-type, and G-type. G-type and L-type LecRKs are activated by PAMP signaling perception, which triggers PTI to HPs. G-type LecRKs Pi-d2 and OsLecRK have been found to trigger plant defense against rice blast and leaf blight, as well as activate various signal responses in plant innate immunity (Li et al., 2015). Among them, the OsLecRK mutant is more susceptible to *Magnaporthe grisea* infection than the wild type, with a reduction in mRNA levels of *PR1*, *LOX2*, and *CHS* defense-related genes (Cheng et al., 2013). Similar to LRR receptors, G-type SBP1 and SBP2 can specifically activate immunity by positively regulating the interaction between RLP23 receptors and BAK1 co-receptors (Bao et al., 2023). G-type LecRK LORE was identified as a target site for the effector HopAO1 of *P. syringae*. During the initial stages of infection, LORE detects bacterial lipopolysaccharides and triggers autophosphorylation to activate the immune response. In the advanced stage of infection, HopAO1 interacts with LORE within host cells, leading to the dephosphorylation of LORE. This effectively suppresses the immune response and makes the host more susceptible to the infection (Chen et al., 2017; Luo et al., 2020). HPs that secrete various molecules recognized by the same host PRR receptors lead to diverse plant immune reactions. Phytohormones play a vital role in plant-pathogen interactions, such as JA, which activate plant defense responses against pathogens. The G-type Lec-RLK FaMBL1 from strawberry can bind to mannose from the cell wall of *Colletotrichum fioriniae*. Overexpression of FaMBL1 leads to a reduction in JA content (Ma et al., 2023). The L-types of LecRK-IX.1, LecRK-IX.2, and LecRK-I.9 in *Arabidopsis* are considered to have defense responses against *Phytophthora*. Overexpression of LecRK-IX.2 phosphorylates RBOHD to enhance ROS production and SA accumulation in PTI response (Wang Y. et al., 2015; Wang Y. et al., 2016; Luo et al., 2017). Similar to LecRK-IX.2, LecRK-I.9 is another L-type LecRK in *Arabidopsis*, known as DORN1, which also confers plant resistance to *P. syringae* DC3000 and *Phytophthor*a resistance (Balague et al., 2017; Sun Y. L. et al., 2020). DORN1 recognizes extracellular ATP signals and directly phosphorylates RBOHD. This induces Ca2^+^ influx, MAPK activation, ROS accumulation, and defense gene expression, and host stomatal closure to restrict HPs invasion (Luo et al., 2017; Wang et al., 2018). These studies indicate that LecRLKs are involved in PTI, ETI, SA, and JA signaling pathways and could enhance plant defense against HPs. In molecular breeding, manipulation of one pathway may impact other signaling responses, due to the interconnected nature of these pathways.

## 4. Intracellular recognition receptors

The NLRs perceive effector proteins in host cells to activate an ETI immune response against these pathogens (Barragan and Weigel, 2021). It is a significant member of the plant resistance R protein family with a conserved modular structure consisting of a central NB-ARC domain (nucleotide-binding adapter, APAF-1, R protein, and CED-4), a C-terminal leucine-rich repeat (LRR) domain, and a unique N-terminal domain (Duxbury et al., 2021). Based on their variable N-terminal structure, it is predominantly categorized as either the coil-coil (CC) type or Toll/interleukin-1 receptor type (TIR), known as CNLs and TNLs (Monteiro and Nishimura, 2018; Yuan et al., 2021a; Maruta et al., 2022). Typically, the CC or TIR in the N-terminal domain is considered the signaling domain. The central NB-ARC domain acts as a molecular switch that regulates the binding or hydrolysis of ADP or ATP to determine the signaling state of NLR. LRR domains may play a significant role in ligand recognition and regulatory activity (Jubic et al., 2019; Ma et al., 2020). NLRs often exist in an inactive state when not infected by pathogens due to various intramolecular and intermolecular interactions. Upon recognition of hemibiotrophic effectors, these interactions are disrupted, which activates the NLR to trigger programmed cell death (Sun Y. et al., 2020).

Nucleotide-binding and leucine-rich repeat receptors recognize pathogen effectors through various strategies, including direct recognition, indirect recognition, and paired NLR recognition (Kroj et al., 2016). Some NLRs recognize effector protein patterns directly, which are extensively characterized. HPs are mainly recognized by NLR receptors indirectly. The indirect interaction between ligands and receptors is well-described in the “guard” and “decoy” recognition models (van der Hoorn and Kamoun, 2008). Other effector targets, including transcriptional regulators, molecular chaperones, and ubiquitin ligases, could serve as potential “guards” or “decoys.” These effector targets regulate transcriptional reprogramming and host protein stability, respectively (Sun Y. et al., 2020; Duxbury et al., 2021). There is also a small portion of plant NLR that includes an additional “integrated” domain (ID). This ID recognizes HPs through integrated decoy patterns and activates downstream immune responses (Sarris et al., 2016). The strategies used to activate immunity by various interactions between NLRs and different HPs are discussed in detail below (Figure 1; Table 2).

**Table 2:** Overview of intracellular recognition receptors and the outcome of interactions with typical HPs to mediate immune results.

Organism	NLR	Type	Pathogen	Effector	Effector target protein	Recognition domain	Evidence	Outcome
Rice	Pi-ta	CNL	*M. oryzae*	AvrPi-ta	None	None	Y2H	Resistance
Rice	Pikp	CNL	*M. oryzae*	AVR-PikD	None	CC	Y2H, BiFC, pull-down	Resistance
Rice	Pikm	CNL	*M. oryzae*	AVR-PikD, AVR-PikE, AVR-PikA	None	CC	Y2H, co-IP	Resistance
*Arabidopsis*	RPM1	CNL	*P. syringae*	AvrRpm1, AvrB	RIN4	CC, FL	Y2H, co-IP	Resistance
*Arabidopsis*	RPS2	CNL	*P. syringae*	AvrRpt2	RIN4	CC, FL	Co-IP	Resistance
*Arabidopsis*	RPS5	CNL	*P. syringae*	AvrPphB	PBS1	CC, FL	Co-IP	Resistance
*Arabidopsis*	ZAR1	CNL	*P. syringae*	AvrHopZ1a	ZED1	CC, CC-NB, FL	Y2H, BiFC	Resistance
*Arabidopsis*	ZAR1	CNL	*P. syringae*	HopF2a	ZRK3	FL	Co-IP	Resistance
*Arabidopsis*	ZAR1	CNL	*P. syringae*	AvrHopZ1a	SZE1, 2	FL	BiFC, co-IP, pull-down	Resistance
*Arabidopsis*	RPM1	CNL	*P. syringae*	AvrRpm1, AvrB	HSP90.2	FL	Co-IP	Resistance
*Arabidopsis*	SNC1	TNL	*P. syringae*	None	HSP90.3	FL	Co-IP	Resistance
*Arabidopsis*	None	CNL	*P. syringae*	HopBF1	HSP90	NB	IP-MS	Susceptibility
*Arabidopsis*	SNC1	TNL	*P. syringae*	None	CPR1	FL	Co-IP, pull-down	Susceptibility
*Arabidopsis*	RPS2	CNL	*P. syringae*	None	CPR1	FL	Co-IP, pull-down	Susceptibility
*Arabidopsis*	SNC1	TNL	*P. syringae*	None	MUSE3	FL	Co-IP	Resistance
Rice	PigmR	CNL	*M. oryzae*	None	PIBP1	CC, FL	Y2H, SLC, BiFC, co-IP, pull-down	Resistance
Rice	Pi9	CNL	*M. oryzae*	None	PIBP2	CC	Y2H, SLC	Resistance
Rice	Piz-t	CNL	*M. oryzae*	None	PIBP1	CC	Y2H, SLC	Resistance
Rice	Piz-t	CNL	*M. oryzae*	AvrPiz-t	APIP5	NT, FL	SLC, pull-down	Resistance
Rice	Pb1	CNL	*M. oryzae*	None	WRKY45	CC, FL	Y2H, SLC, co-IP, pull-down	Resistance
*Arabidopsis*	RPS4	TNL	*P. syringae*	AvrRps4	bHLH84	FL	Co-IP	Resistance
Rice	Piz-t	CNL	*M. oryzae*	AvrPiz-t	APIP5	None	Co-IP, pull-down, BiFC, Y2H	Resistance
*Arabidopsis*	RPS4	CNL	*P. syringae*	AvrRps4	EDS1	FL	BiFC, co-IP	Resistance
*Arabidopsis*	RPS4	TNL	*P. syringae*	AvrRps4	RRS1	TIR, FL	Y2H, co-IP, pull-down	Resistance
Rice	RGA4	CNL	*M. oryzae*	AVR1-CO39, AVR-Pia	RGA5	CC, FL	Y2H, co-IP	Resistance
Rice	Pik-2	CNL	*M. oryzae*	AvrPik	Pik-1	CC	Y2H, BiFC, pull-down	Resistance

^a^HP, Hemibiotrophic pathogen; CNL, CC-NLR; TNL, TIR-NLR; CC, Coiled-coil; NB, Nucleotide binding; NT, N-terminus; FL, Full length; TIR, Toll/interleukin 1 receptor (TIR) domain; Y2H, Yeast two-hybrid; BiFC, Bimolecular fluorescence complementation; Co-IP, Coimmunoprecipitation; SLC, Split luciferase complementation; IP-MS, Immunoprecipitation, and mass spectrometry; *P. syringae*, *Pseudomonas syringae*; and *M. oryzae*, *Magnaporthe oryzae*. ^b^None indicates that no data are available. ^C^This table is not a detailed list of all plant receptors for detecting HPs.

### 4.1. NLR directly senses HPs to confer plant immunity

Most of the proteins encoded by R genes in each plant genome are NLRs. NLRs directly or indirectly recognize effectors secreted by HPs and activate ETI response (Barragan and Weigel, 2021). Direct interaction between hemibiotrophic effectors and plant NLRs is the most intuitive and simple method. Most CNLs act as effector receptors, called sensor CNL. For example, plant CNLs detect their cognate effectors via direct interactions. Rice NLR Pi-ta binds to the *M. oryzae* effector AVR-Pita (Jia et al., 2000). Intriguingly, the CNL protein encoded by the *Pik* allele in rice can also perceive multiple AVR-Pik effectors of *M. oryzae* via physical interaction. Pikm can recognize three AvrPik effectors of *M. oryzae*, while Pikp can recognize only one (De la Concepcion et al., 2018).

### 4.2. NLRs indirectly sense HPs to confer plant immunity

Hemibiotrophic pathogens are mainly recognized by NLR receptors indirectly. Two recognition models can well describe the indirect interaction between ligands and receptors, namely the “guard” model and the “decoy” model (van der Hoorn and Kamoun, 2008). Modification of guards or decoys by effectors may cause changes in the conformation of NLRs, which leads to the activation of ETI (Schreiber et al., 2016). Since many effectors targeted by NLRs are unknown, and it is often unclear whether these effector targets are guardees or decoys (Kapos et al., 2019). The “guard” model suggests that NLR proteins monitor the integrity of target proteins in plant cells and activate immune responses upon perturbation or modification by pathogen effectors. For example, *P. syringae* effectors AvrRpm1, AvrB, and AvrRpt2 specifically target the guardee protein RPM1-interacting 4 (RIN4), while CNLs RPM1 and RPS2 monitor RIN4 steric hindrance or post-translational modification to exert disease resistance (Day et al., 2005; El Kasmi et al., 2017). In soybean, CNL RPG1-B monitors the homolog RIN4 in a comparable way (Selote and Kachroo, 2010). Another well-studied example is that of the guardee CNL RPS5 monitors the host target kinase PBS1, where the *P. syringae* effector protease AvrPphB cleaves PBS1 to activate RPS5-mediated immunity (Ade et al., 2007). It is generally established that all kinases and pseudokinases serve as “guards” or “decoys,” and interact with CNLs, but not with TNLs. Decoy proteins have no defined biological, cellular, or physiological role in host defense. Instead, they imitate toxic targets to activate the host surveillance system and detect effector molecules. Decoys probably evolved by duplicating ancestral guardians (van Wersch et al., 2020). As a decoy protein, the pseudokinase RLCK XII family ZED1 interacts with the acetyltransferase HopZ1a effector secreted by *P. syringae*. ZED1 is acetylated to activate CNL ZAR1 (Wang G. X. et al., 2015). Recent research revealed that ZED1 forms a complex with ZAR1 after acetylation by HopZ1a, triggering the assembly of higher-order complexes in plants that form a resistosome similar to the ZAR1-RKS1-PBL2^UMP^ complex (Hu et al., 2020). Other RLCKs are targeted by HopZ1a and are recognized by the ZAR1-ZED1 complex. RKS1 performs an adapter function similar to ZED1 (Bastedo et al., 2019). The pseudokinase ZRK3 and the RLCK family SZE1 and SZE2 also bind to ZAR1 (Liu et al., 2019). It appears that indirect interactions may expand the capacity of certain plant immune receptors to detect additional pathogen effectors. Furthermore, indirect interactions may offer more avenues to enhance pathogen control by regulating receptors.

Plant NLRs’ assembly, activity, and stability are tightly regulated to ensure appropriate host defense responses against HPs. Studies have shown that molecular chaperones and ubiquitin ligases are essential for NLRs’ assembly, activity, and stability (Duxbury et al., 2021; Huang et al., 2021). There are several chaperones in the HSP90 family that play a role in NLR-mediated defenses in *Arabidopsis*. HSP90.2 interacts with CNL RPM1 to strengthen RPM1 protein stability. HSP90.3 interacts with TNL SNC1 to negatively regulate SNC1 accumulation (Hubert et al., 2003; Huang S. et al., 2014). Recent studies have shown that the *P. syringae* effector HopBF1 phosphorylates HSP90 to trigger hypersensitivity in plants. This finding uncovers a previously unidentified mechanism by which hemibiotrophic bacteria regulate host immunity (Lopez et al., 2019). And HSP90 may assist the suppressor of G-two allele of SKP1 (SGT1) in forming the Skp1-Cul1-F-box (SCF) E3 ubiquitin ligase complex, which targets immune receptors for degradation. This process is critical for maintaining appropriate levels of immune receptor proteins to avoid autoimmunity. Similarly, ubiquitin ligases interact with NLRs to help regulate their levels without pathogen infection. Upon *P. syringae* infection, the proteasome effector destroys the E3 ligase F-box protein CPR1, interacts with *Arabidopsis* TNL SNC1 and CNL RPS2, and reduces their protein accumulation, thus inducing a defense response (Gou et al., 2012). Knocking down the E4 ligase MUSE3 in *Arabidopsis* causes increased levels of TNL SNC1 and CNL RPS2. Overexpression of MUSE3 and CPR1 enhanced polyubiquitination and protein degradation of these immune receptors (Huang Y. et al., 2014). Host molecular chaperones and ubiquitin indirectly control NLR activity and stability to modulate immune responses when various HP infect the host. This suggests that NLR activity and homeostasis are critical for plant disease resistance.

Transcriptional reprogramming is a frequent occurrence in plant immunity that involves numerous transcriptional regulators. The coordination and nuclear localization of NLRs and immune transcription factors in the transcriptional machinery is crucial to selectively activate plant defense genes during HPs infection. The transcription factor RRM shows CNL-dependent nuclear localization and is unaffected by HPs. RRM binds to the CNL encoded by the resistance genes *PigmR*, *Pi9,* and *Piz-t*, and establishes a direct link between transcriptional activation of the immune response and NLR-mediated pathogen perception by directly binding to the A/T-rich cis-element DNA in the target gene (Zhai et al., 2019). This is in contrast to the nuclear localization of the constitutive transcription factor WRKY45. This transcription factor induces resistance through the SA signaling pathway regulated by the ubiquitin-proteasome system. The CNL protein encoded by the rice blast resistance gene *Pb1* interacts with WRKY45 in the nucleus to regulate broad-spectrum resistance to *M. oryzae* (Inoue et al., 2013). EDS1 regulates the defense signaling pathway. The interactions between *Arabidopsis* NLR RSP4 and EDS1 result in similar but distinct nuclear NLR-dependent translocations. The RPS4 and EDS1 complex is predominantly located in the cytoplasm, but can also be observed in the nucleus during homeostasis or upon AvrRPS4 infection. Additionally, RPS4 binds to EDS1 in the cytoplasm without relying on another NLR RRS1. Instead, when RRS1 is present, an RPS4-EDS1 complex is observed in the nucleus. This suggests the existence of pre- and post-activation states for the nuclear localization of RPS4, RRS1 and EDS1 complexes. The RPS4-EDS1 binding in the nucleus may be unaffected by HP effectors such as AvrRps4 in the presence of RRS1 (Wang R. Y. et al., 2016). It has also been shown that the effector Avrpiz-t from *M. oryzae* interacts with the bzip-type transcription factor APIP5 in the cytoplasm, inhibiting its transcriptional activity and protein accumulation during the necrotic stage. Additionally, the rice NLR Piz-t inhibits plant ETI necrosis by interacting with APIP5 (Wang R. Y. et al., 2016). These showing how the host utilizes transcription factors as imitation substances for effectors to prevent host immune responses induced by various HPs.

### 4.3. Paired NLRs sense HPs to confer plant immunity

Many NLRs paired with additional domains or motifs are also critical for plant protection against HPs compared to regular NLRs. The paired NLR contains one NLR with an ID at its C-terminus that mimics the virulence target of an effector protein, and thus acts as a sensor for detecting effector proteins. Another NLR acts as a classical executive NLR that performs signal transduction functions (Grund et al., 2019). Some NLR gene pairs are frequently close in the same locus on the chromosome. These pairs share a promoter. The two genes that encode RPS4 and RRS1 are located adjacent to each other and are arranged in opposite directions. This suggests they may co-regulate transcription, with an interval of approximately 300 bp between them (Narusaka et al., 2009). RRS1 has an extra structural domain called WRKY at the C-terminus. RPS4 and RRSI together form a heterodimeric complex that recognizes the effector AvrRps4 and confers resistance to *P. syringae* (Guo et al., 2020). In rice, the C-terminus of the NLR-paired RGA5 contains an HMA structural domain that acts as an ID that interacts with the effectors AVR1-CO39 and AVR-Pia in *M. oryzae*. The NLR-paired RGA4 acts as an NLR executor that induces robust HR in tobacco leaves (Cesari et al., 2013; De la Concepcion et al., 2021). The co-expression of Pikp-1, Pikp-2, and *M. oryzae* effector AVR-PikD, where Pikp-1 possesses an HMA, and Pikp-2 conducts signal transduction, induces a significant HR in tobacco (Maqbool et al., 2015; Cesari et al., 2022). Guardees or decoys allows plants to detect a wide range of pathogen effectors with a relatively small repertoire of NLRs (Frailie and Innes, 2021; Liu et al., 2021). In the RPS4/RRS1 and RGA4/RGA5 heterodimers, one NLR is involved in effector identification while the other is involved in defense signaling. Understanding the extent of heterodimerization in plant NLRs is crucial in gaining insights into plant NLR evolution and diversity. Moreover, pairing NLR sensors and actuators in plant design could improve effector recognition specificity and resistance profiles.

## 5. Conclusion and perspective

Plant diseases caused by phytopathogens are a major threat to global food security and can lead to significant economic losses, such as HPs. Notably, *M. oryzae*, *Colletotrichum* spp., and *P. syringae* are considered important HPs due to their ease of cultivation, genetic modification, and typical hemibiotrophic characteristics (Doehlemann et al., 2017). Therefore, they are commonly utilized as models for studying plant immunity activation by HPs. Several other species from broad genera are also classified as HPs, including significant plant pathogens like *Fusarium* (Ma et al., 2013)*, Verticillium* (Fradin and Thomma, 2006)*, Mycosphaerella* (Churchill, 2011), and others. All of these species have an asymptomatic stage of varying lengths. In most cases, they do not develop into typical biotrophic specialized organs and do not make close contact with the host cells. Therefore, the pathogenic lifestyle of HPs comprises asymptomatic, quiescent, latent or endogenous stages and requires different forms of control. To effectively manage plant diseases caused by HPs, it is essential to understand the interaction between HPs and host plants, as well as their strategies for activating plant immunity. Currently, genetic control of plant diseases aims to improve plant resistance. This is achieved through techniques like genome editing, which targets specific genes, and resistance gene enrichment sequencing. Plant disease resistance is mostly determined by genes that have receptors detect when pathogens enter the plant and trigger immune responses. In this review, we discuss plant cell surface PRR and intracellular NLR immunoreceptors that detect various HP and initiate appropriate immune responses. Furthermore, we discuss effector target proteins, including transcription factors, ubiquitin ligases, and molecular chaperones. These proteins could potentially function as models of “guards” and “decoys” for the indirect molecular immunity against HP in the ongoing arms race between plants and pathogens. Over time, a complex recognition system has developed between the immune receptors and HP. By summarizing the interaction between HPs and plant immune receptors that trigger host immune responses, it was found that PAMPs may be conserved and prevalent in various microorganisms, and the cell-surface PRR complexes have similar co-receptors, including BAK1, SOBIR1, or CERK1. By combining genomics, transcriptomics, effectomics, and high-throughput phenotypic analysis, it is anticipated that that crops will achieve better and sustainable protection against various diseases through the utilization of stacked plant immune receptors. For example, multiple plant immune receptors may be designed to recognize the same type of pathogen, or they may be designed for different types of pathogens, such as oomycetes and fungi. We may discover novel defense mechanisms and corresponding pathogen factors. This results in improved disease management and control.

However, it is unclear how these simple conceptual models allow PRRs and NLRs to adapt to the virtually unlimited of immune signaling space. Moreover, the *Agrobacterium* transient assay is useful for enhancing analysis of plant receptor function in solanaceous hosts. However, it might not work well for studying receptor function in soybean or other monocotyledonous plants. This difficulty has also been a major constraint in finding different pathogen effector proteins. Plant PTI and ETI signaling systems may appear simple, but they are rather complex. One immune receptor like EDSI could regulate a single target protein of the host and may be targeted by multiple effectors like RIN4 (Sun Y. L. et al., 2020). Each signal directly or indirectly coordinates with each other to jointly regulate plant immunity, growth and development. Elimination of host susceptibility to HPs through gene editing techniques poses potential risks. New susceptibility genes may be introduced while the desired traits are being engineered for transfer to other species. A recent study of cell trajectory analysis of based on single-cell omics technology found that, at the early stages of immune cell trajectory, the progression of disease from the immune state to the susceptible state is a continuous process (Zhu et al., 2023). In the future, advancements in spatial omics with high resolution, next-generation sequencing technology, and new bioinformatics algorithms and pipelines will provide new genome-wide data for HPs. This will enable researchers to gain greater insight into intricate plant immune responses and their dynamic interactions with pathogens in specific spatial contexts. However, with so many candidate effector genes or genomes, it is unclear how HP effectors shift from biotrophy to necrotrophy, manipulate host targets, and interfere with plant immune activation.

## Author contributions

DZ, GZ, and JL contributed to the conception of the manuscript. DZ wrote the original draft. XZC, XGC, and YX provided editing assistance. All authors contributed to the article and approved the submitted version.
